# Phytochemical and Bioactive Analysis of Extracted Brown Macroalgae (*Dictyota implexa*) Collected in Vietnam

**DOI:** 10.1155/bri/9461117

**Published:** 2025-03-29

**Authors:** Nguyen Ngoc Trang Thuy, Tran Thanh Men

**Affiliations:** ^1^Faculty of Food Technology Biotechnology and Chemical Technology, Can Tho University of Technology, Can Tho, Vietnam; ^2^Institute of Food and Biotechnology, Can Tho University, Can Tho, Vietnam; ^3^College of Natural Sciences, Can Tho University, Can Tho, Vietnam

**Keywords:** anti-inflammatory, antimicroorganism, antioxidant, cytotoxicity, *Dictyota implexa*

## Abstract

Brown algae are considered a marine algae resource for human health. This study investigated ethanol extract's chemical composition and biological activity from brown algae *Dictyota implexa.* The extract from *D. implexa* was examined for total contents of quercetin, tannic acid, phenolic, flavonoid, polysaccharides, agar, and fucoidan, and the antioxidant, anti-inflammatory, antibacterial, cytotoxic, and α-amylase inhibitory activities of the crude extract were determined. Results revealed the presence of a source of phenolic (85.95 ± 1.21 mg GAE/g of the sample), flavonoid (245.6 ± 2.83 mg QE/g of the sample), and tannin (172.179 mg/g DW) compounds in the extract. Evaluating antioxidant activity proved the ethanol extract of *D. implexa* possessed the highest activity on two testing methods of DPPH scavenging capacity and reducing power. Besides, the anti-inflammatory activity was potent in the extract with an IC_50_ value of 9.95 ± 1.51 μg/mL. Concerning antimicrobial activities, the ethanol extract of *D. implexa* (70 mg/mL) showed potential inhibitory ability against *E. coli* and *B. cereus*. Moreover, the algal extract displayed cytotoxic activity against HeLa cells and inhibited α-amylase activity with an IC_50_ value of 276.82 μg/mL. The current findings demonstrated that exploring novel natural resources offers a promising avenue for advancements in human health and economic well-being.

## 1. Introduction

Seaweeds (or macroalgae), primarily located in marine environments [1], can be harvested from the wild or farmed extensively along coastlines. They thrive by utilizing the ocean's natural nutrient supply [2]. Seaweeds are a rich source of essential minerals and biochemicals, but the exact composition varies depending on the specific type of seaweed, where it grows, the time of year, and its physiological state [3, 4]. Different types of seaweed have different bioactive compounds and functions [5]. Seaweeds are generally categorized into three main groups: red algae (Rhodophyta), green algae (Chlorophyta), and brown algae (Ochrophyta, specifically the class Phaeophyceae) [6, 7]. Brown seaweed *Dictyota implexa* belongs to the phylum Heterokontophyta, class Phaeophyceae, family Dictyotaceae, genus *Dictyota*, and species *implexa*. Greenish-yellow seaweed grows in tangles, 5–8-cm high or more, attached by pseudoroots growing from the base. The belt shape is narrow, twisted, 0.3–1.5-mm wide, branched in double or irregular forks, branch angles are slightly rounded or pointed, tops are split in two, and branch tips are obtuse or slightly pointed. Phaeophyceae algae arise primarily from the emergence of carotenoid pigments called fucoxanthins [8]. Notably, these algae are gaining recognition as a significant source of bioactive compounds, offering potential health benefits for humans [9].

For example, marine algae are attracting considerable interest for their remarkable features of natural antibacterials, with extracts and active constituents from various species demonstrating efficacy against Gram-positive and Gram-negative bacteria [10, 11]. The chemical composition of these antimicrobial compounds is remarkably diverse [12]. However, a crucial issue needs to be concerned is that environmental factors such as season, habitat, and collection age can impact the metabolic profile of marine algae, ultimately damaging the nature and concentration of these bioactive constituents [13]. The development of antibiotics is considered a landmark achievement in modern medicine, revolutionizing our ability to control infectious diseases. Marine algae offer a potential avenue for discovering novel antibiotics, potentially complementing existing therapies [14].

In addition to primary metabolites, macroalgae also produce secondary metabolites, which are compounds not directly involved in the organism's basic metabolic processes and are often unique to specific species or groups of algae. Examples of these secondary metabolites include agar, alginate, fucoidan, ulvan, laminarin, starch, cellulose, pectin, and carrageenan [15]. Because algae produce a wide variety of these compounds, they are considered a valuable natural source of bioactive substances [16].

*D. implexa* is found along numerous Vietnamese coastlines, including Kien Giang Province and its Hon Son Island. A study by Do and Do [17] suggested that the Kien Giang coast's abundant seaweed diversity is linked to its large area and varied ecosystems, encompassing coastal zones, tidal areas, and coral reefs. The same study also proposed that coral decline may be contributing to seaweed growth by providing dead coral as a substrate [17]. While Vietnamese seaweeds are known to be a source of beneficial bioactive compounds, they have not been studied extensively. This research gap highlights a large, unexplored potential, especially given the promising health benefits seen in similar seaweeds. Therefore, this study focuses on *D. implexa* to investigate its potential bioactivity. Generally, this work aims to quantify the secondary metabolites present in brown macroalgae species *D. implexa* collected from Hon Son Island in Kien Giang, Vietnam. By evaluating the antioxidant, anti-inflammatory, antimicrobial, cytotoxic, and antidiabetic properties of extracts from this macroalgae, the study seeks its potential applications in the pharmaceutical, medicinal, and nutritional fields. Our research is likely to interest seaweed researchers investigating brown seaweed as a sustainable source of valuable compounds. These compounds could boost Vietnam's economy and improve its environment.

## 2. Materials and Methods

### 2.1. Chemicals and Reagents

Folin–Ciocalteu reagent (Merck), sulfuric acid (Xilong Scientific, China), quercetin (Xilong Scientific, China), diclofenac natri (Xilong Scientific, China), gallic acid (Xilong Scientific, China), agar (Terko, Vietnam), ethanol 96° (Ngan Huong, Vietnam), ethanol 99.5° (Xilong Scientific, China), methanol (Xilong Scientific, China), 1,1-diphenyl-2-picrylhydrazyl (DPPH) (Tokyo chemical industry, Japan), and other chemicals were of analytical grade made in China.

### 2.2. Collection of Algal Material

Brown seaweed *D*. *implexa* was collected from Hon Son Island of Kien Giang coast, Vietnam. The time collection of algae was in March 2023, and the seaweed was collected with all parts of its organism by hand. The seaweeds were identified at the Department of Biology, Faculty of Natural Sciences, Can Tho University, according to the Vietnamese seaweed classification system and then using the algae base website to confirm their identification. *D. implexa* is greenish-yellow algae, growing in tangled clumps, 5–8-cm high or more, attached by false roots growing from the base. The algal sample is stored in the laboratory of the Department of Biology, Faculty of Natural Sciences, Can Tho University, and the voucher specimen number is B.imp.3.23.

The collected seaweed was thoroughly washed with seawater and placed in a zip bag before transferring to the laboratory. It was then kept in cool condition for further experiments. Fresh algae was air-dried in the shade at room temperature and then in the oven at 38 ± 2°C. The dried seaweed was cut into small pieces (1–2 cm) to be ground with an electric mixer. The algae sample was put in closed plastic bags and stored in the refrigerator for further use.

### 2.3. Extract Preparation

The classical approach to prepare the extract was macerating dry algae (25 g) in 250 mL of 96^o^ ethanol. It was then extracted at room temperature for 24 h. Whatman no. 41 was used to filter the filtrate. After placing it in a glass container, the filtrate was evaporated at 60°C by a rotary evaporator. The completion could not be reached until the concentrated extract was obtained. The extracted sample was stored in the refrigerator for further application.

### 2.4. HPLC Analysis

#### 2.4.1. Determination of Quercetin and Tannic Acid Content

The experiment to determine the quercetin and tannic acid content closely resembled the method of Ang et al., with some modifications. A reverse-phase HPLC assay was made using isocratic elution with a flow rate of 1.3 mL/minute, a column temperature of 35°C, a mobile phase of acetonitrile, 2% v/v acetic acid (pH 2.60) (40%: 60% v/v) and a detection wavelength of 370 nm. The injection volume was 15 μL of each solution. Statistical significance was acquired and analyzed through LC-Solution Software. Solvents and distilled water were filtered through a 0.45-μM nylon membrane using a set of glass bottles with a vacuum pump [18].

### 2.5. FTIR Analysis

In an attempt to analyze the FTIR, a mixture including 2 mg of the algal sample and 200 mg KBr (FTIR grade) was pressed into a homogeneous pellet. The pellet was immediately put into the sample holder FTIR Spectrophotometer Prestige-21. The spectra bands were observed and recorded in the range of 4000–400 cm^−1^ [19, 20].

### 2.6. Quantitative Analyses

#### 2.6.1. Total Phenolic Content

The method to determine the total phenolic content of the *D. implexa* extract was inspired by the Folin–Ciocalteu method of Sari et al. [21] with some modifications. A mixture composing 0.5 mL extract solution and 2 mL Folin–Ciocalteu 10% was shaken for 1 min in the test tube. It was then taken to incubate at 20°C–25°C until 4 min. Continue to add 1.5 mL of sodium carbonate solvent (10%) and shake it for 1 min. The resulting mixture was subsequently incubated at room temperature. The process could not be completed until 2 h. The absorbance of the mixture was measured using a spectrophotometer at 760-nm wavelength and compared to the gallic acid calibration curve (0–40 μg/mL).

#### 2.6.2. Total Flavonoid Content (TFC)

The TFC of the D. *implexa* extract was determined using the variation of Bag et al. method with some modifications. A concentration of 0.5 mg/mL (1 mL) of the extract solution was mixed with 1 mL distilled water and 200 μL NaNO_2_ 5%. The resulting solution was then shaken and kept stable for 5 min. The mixture was continued to be added 200 μL of AlCl_3_ solvent (10%) and shaken. The procedure was kept moving on by incubating the mixture at room temperature until 6 min. Then, 2000 μL of NaOH (1M) and distilled water were added to the mixture to make up 5 mL. The absorbance of the mixture was measured using a spectrophotometer at 510-nm wavelength and compared to the quercetin calibration curve (0–120 μg/mL) [22].

#### 2.6.3. Estimation of Polysaccharide Content

The content of polysaccharides in the algal extract was determined by the phenol–sulfuric acid method suggested by Khamlue et al. with some modifications. 1 mL of the algal extract (20 μg/mL) was pipetted into a test tube, and 0.5 mL of 5% phenol solutions was added. Then, 2.5 mL of concentrated sulfuric acids was added rapidly. The tubes were shaken and placed for 30 min at room temperature. The absorbance of the mixture was measured using a spectrophotometer at 490-nm wavelength and compared to the glucose calibration curve (0–100 μg/mL) [23].

#### 2.6.4. Estimation of Agar Content

The estimation of the agar content of *D*. *implexa* was verified as described in the method of Villanueva et al. (2010) with some modifications. 10 g dry sample was prepared to be soaked in a 100 mL of alkali solution (6% at 95°C for 30 min). The alkali solution was discarded. The algae material was washed thoroughly with fresh water before being soaked in 100 mL citric acid for 30 min at room temperature. The citric acid solution was discarded, and the seaweed was replicatedly washed with fresh water. Keep soaking seaweed in 100 mL acetic acid (10%) for 30 min at 95°C. The experiment was stayed proceeding by filtering the mixture by a filter paper. Agar was collected through the freeze-thawing method to be oven-dried at 60°C to obtain the agar extract [24]. The agar yield (%) was recorded as the percent of dry matter.

#### 2.6.5. Estimation of Fucoidan Content

The estimation of the fucoidan content of *D*. *implexa* followed the method of Palanisamy et al. (2017) with some modifications. The procedure was started by soaking 25 g in 250 mL ethanol (85%). The mixture was stirred at room temperature for 12 h before subjecting to be centrifuged at 6000 rpm for 10 min. The supernatant was discarded and the residue was allowed to be dried at room temperature to obtain Powder A. The process was continued by taking 5 g of Powder A to mix in 100 mL of distilled water. The mixture was stirred for 1 h at 65°C. Subsequently, it was centrifuged again at 6000 rpm for 10 min. Then, the supernatant was collected and mixed with 1% CaCl_2_. The solution was maintained overnight at 4°C to precipitate alginic acid. Keep centrifugating the solution at 6000 rpm for 10 min. The collected supernatant was mixed with 99% ethanol to reach the final ethanol concentration of 30% and kept at 4°C for 2 h. After 2 h incubation, the solution was once again centrifuged at 6000 rpm for 10 min to obtain the supernatant. Then, 99% ethanol was added to the supernatant to make up the concentration of 70%. The solution was stored for 2 h at 4°C and then centrifuged at 6000 rpm for 10 min to obtain the fucoidan. The fucoidan yield (%) was calculated as the percentage of algal dry weight (% dry weight) [25].

### 2.7. Antioxidant Activity Assay

#### 2.7.1. DPPH Radical Scavenging Activity

A solution of DPPH with a concentration of 6 × 10^−4^ M was prepared in methanol. DPPH radical scavenging activities of the D. *implexa* extract was determined using a variation of the method of Sharma et al. with some modifications. Briefly, ethanol extract solution of D. *implexa* at 1000–8000 μg/mL (0.5 mL) was mixed with prepared DPPH solution and 3 mL methanol. The mixture was shaken vigorously. The reaction tubes, in triplicate, were wrapped in aluminum foil and kept at room temperature for 30 min in the dark [26]. Spectrophotometric measurements were calculated at 517 nm using a Libra S60PC spectrophotometer. The data are expressed as mean ± SD.

The measurement of activity to scavenge the free radicals was loosely based on a decrease in the absorbance of DPPH and calculated as follows:

(1) $$\begin{array}{c} s\text{cavenging effect}\left(\%\right)=\frac{a\text{bsorbance of control}-a\text{bsorbance of sample}}{a\text{bsorbance of control}}\times 100. \end{array}$$

The values of IC_50_ in g/mL were calculated by analyzing the linear regression to represent the concentration of the test samples causing 50% inhibition (IC_50_).

#### 2.7.2. Reducing Power (RP)

In this method, an antioxidant compound forms a colored complex with potassium ferricyanide, trichloroacetic acid, and ferric chloride, which is measured at 700 nm. The antioxidant activity of the *D. implexa* extract was determined according to the method of Alam et al. with modification. Briefly, ethanol extract solution of *D. implexa* with a concentration of 100–2000 μg/mL (0.5 mL) was mixed with 0.5 mL of 0.2 M phosphate buffer (pH 6.6) and 0.5 mL of K_3_Fe (CN)_6_ (1% w/v). The resulting mixture was incubated at 50°C for 20 min, followed by adding 0.5 mL of trichloroacetic acid (10% w/v). The mixture was centrifuged at 3000 rpm for 10 min to collect the upper layer of the solution (0.5 mL) and then mixed with distilled water (0.5 mL) and 0.5 mL of FeCl_3_ (0.1%, w/v). The absorbance was then measured at 700 nm against a blank sample [27].

### 2.8. Inhibition of Protein Denaturation

The protein denaturation method described by Prakash et al. (2013) was adopted and used to evaluate the anti-inflammatory activity of the *D. implexa* extract. Briefly, the reaction mixture containing 2 mL of ethanol extract solution of *D. implexa* (5–100 μg/mL) or reference drug diclofenac sodium (5–100 μg/mL), and 2.8 mL of phosphate-buffered saline (pH 6.4) was mixed with 0.2 mL of bovine serum albumin (BSA) and incubated at 37°C for 15 min. Denaturation was induced by keeping the reaction mixture in a water bath at 70°C for 10 min. After cooling, the absorbance was measured at 660 nm using a Libra S60PC spectrophotometer [28]. The percentage inhibition of protein denaturation was calculated by using the following formula:

(2) $$\begin{array}{c} \%\text{ of inhibition}=100\times \left[\frac{Vt}{\left(Vc-1\right)}\right], \end{array}$$

where *Vt* = the absorbance of test sample and *Vc* = the absorbance of control.

### 2.9. Antibacterial Assay

*Escherichia coli* ATTCC® 25922TM and *Bacillus cereus* ATCC® 10876TM were donated by the Department of Biology, College of Natural Sciences, Can Tho University, Vietnam. The turbidity of the suspension was standardized against 0.5 McFarland using a spectrophotometer at 600-nm wavelength. The bacterial inoculum was 10^8^ cfu·mL^−1^.

Antibacterial activity tests included positive, negative control, and antibacterial activity tests for the algal extract. The positive control test was carried out using tetracycline, and the negative control test used solvents (ethanol and dimethyl sulfoxide [DMSO]). The method applied in this test was the well diffusion test with modification [29]. 25 mL of lactose broth agar was melted, cooled to 55°C, and poured into the assay plate (9 cm in diameter). Then, the mixture was allowed to cool down on a leveled surface. Once the medium had solidified, spread 100 μL of the inoculum evenly over the entire agar surface, ensuring no gaps between streaks. Subsequently, four wells, each 6 mm in diameter, were cut out of the agar, and *D. implexa* extracts with a concentration of 40–70 mg/mL were placed into each well. The samples were incubated for 24 h at 37°C. The distinct zone around the well was a sign of the inhibition zone of bacterial activity. Every experiment was conducted three times. Inhibition zones 15–20 mm were declared as strong, > 11–< 15 mm as moderate, and ≤ 10 mm as weak activities [30].

### 2.10. Cytotoxic Activities

Four cancer cell lines (HT-29, HeLa, Huh7, and HepG2) and a normal cell line (HEK-293) were supplied by Kyoto Institute of Technology, Japan. Cells were cultured with Dulbecco's modified Eagle's medium (DMEM; FUJIFILM-Wako, Japan) supplemented with 10% fetal bovine serum (FBS) and 1% penicillin–streptomycin (PS) in a cell culture cabinet (temperature 37°C; humidity 5% CO_2_). The experiment extract was taken to be dissolved in 5% (v/v) DMSO and diluted with DMEM. Cells were cultured in 96-well plates at a density of 1 × 10^4^ cells/well and incubated for 48 h under the same experimental conditions. The assessment of cell viability was performed by Cell Counting Kit-8 (CCK-8; Dojindo Molecular Technologies Inc., USA). The culture medium was removed in 96 wells after 48 h of incubation. 100 μL of CCK-8 solution in a 10:1 (v/v) ratio was added to each well and incubated for 3 h at 37°C. The measurement of absorbance was taken at 450 nM using a microplate reader (SH-1200, Corona Electric, Japan). Cytotoxicity was shown as the percent inhibition of cell viability in the groups with the extracts or the compound, compared with that of the control group without the extracts.

### 2.11. α-Amylase Inhibitory Activity

The test samples were able to inhibit starch hydrolysis, according to the method of Dai et al. (2012), with adjustments. The reaction mixture included 50 μL of phosphate buffer solution (pH = 7) with 50 μL of sample solution and 50 μL of α-amylase enzyme (3U) incubated at 37°C for 5 min. Then, 50 μL of starch (2 mg/mL) was added to the above mixture and incubated at 37°C for 15 min. Subsequently, 200 μL of concentrated HCl solution was added to stop the reaction. Finally, 300 μL of iodine reagent solution was added to identify the amount of remaining starch after the reaction based on the characteristic blue reaction. The above mixture was measured for the spectral absorbance of the starch–iodine complex at a wavelength of 660 nm. Acarbose was used as a positive control [31].

The activity of α-amylase was calculated as follows:

(3) $$\begin{array}{c} \%\text{ inhibition}=100–\left(\frac{\left(Ao-A1\right)}{\text{Ao}}\times 100\right), \end{array}$$

where *Ao* is the absorbance of control solution and *A*1 is the absorbance of the solution after the reaction.

### 2.12. Statistical Analysis

All experiments were conducted in triplicate (*n* = 3). Data are expressed as mean ± standard deviation (±SD), and error bars in the figures indicate standard deviation. The data were analyzed using Microsoft Excel 2013, and Minitab 17 software used to analyze one-way ANOVA with a 95% confidence level.

## 3. Results and Discussions

### 3.1. Result in HPLC Analysis

**Figure 1:**
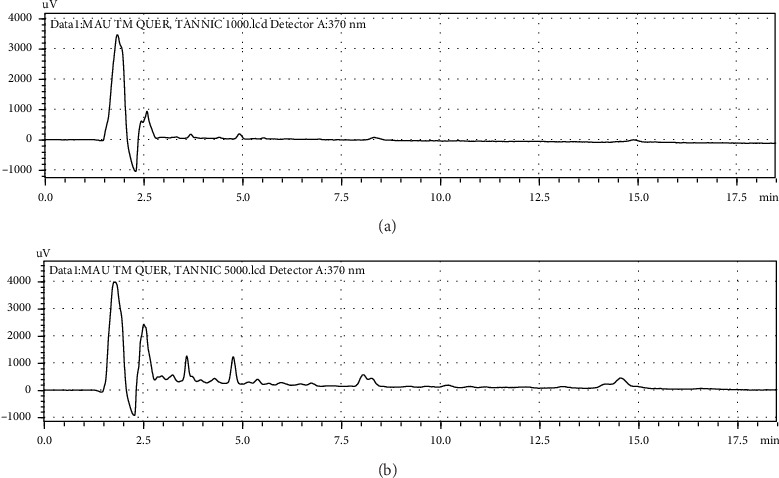
HPLC chromatogram of ethanolic extract of *D. implexa*. (a) *D. implexa* showed quercetin peak at retention time 3.825 min detected at 370 nm and (b) *D. implexa* showed tannic acid peak at retention time 1.833 min detected at 370 nm.

HPLC profile of ethanol extract was examined, and two components, namely, tannic acid and quercetin, should be obtained at distinct retention times (Figure 1 and Table 1). It was remarked that main peaks were eluted with retention times of 1.833 and 3.825 min, respectively, identified at 370 nm. These major compounds were identified as tannic acid and quercetin.

The calibration equation obtained from the calibration curve was *y* = 17907*x* − 864.2 (*R*^2^ = 0.9999) for tannic acid and *y* = 23288*x* − 1000000 (*R*^2^ = 0.9955) for quercetin. Similarly, the HPLC analysis showed that phenol and flavonoid are present in the ethanolic extract of *D. implexa* as the functional constituents. References also proved the findings of the present study, and phytoconstituents such as quercetin and tannic acid in the ethanolic extract of brown algae have indicated the existence of phenolic and flavonoid components in *Turbinaria conoides* by using HPLC [20, 32].

### 3.2. Fourier Transform Infrared (FTIR) Analysis

**Figure 2:**
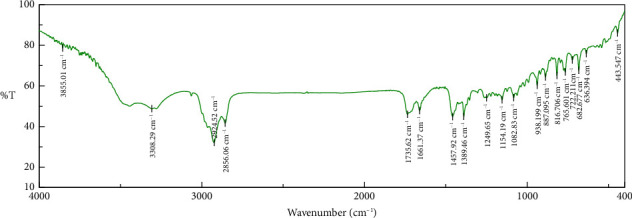
Fourier transform infrared analysis of the *D. implexa* extract.

FTIR analysis was employed to elucidate the chemical composition of the algal biomass sample. As depicted in Figure 2, peaks observed over the 3600 cm^−1^ region across the sample are tentatively assigned to O–H stretching vibrations associated with hydroxyl functional groups in carboxylic acids, phenolics, and alcohols. The band observed in the 2800–3000 cm^−1^ range can be attributed to both C–H and =C–H stretching vibrations, indicative of lipid and carbohydrate content within the algal sample. Additionally, this region may encompass N–H stretching vibrations originating from proteins. A significant band at 1709–1583 cm^−1^ corresponds to C=O stretching vibrations of carbonyl groups in lipids and specific polysaccharides, such as alginate, laminarin, and fucoidan. Furthermore, the presence of a peak within the 1099–1356 cm^−1^ wavenumber range suggests potential breakdown and leaching of polysaccharides. The band observed at 980–1072 cm^−1^ is tentatively assigned to C–O–C stretching vibrations in polysaccharides. The sharp signals in the sample at 887.095 and 816.706 cm^−1^ may be due to the out-of-plane deformation C–H bending mode of glucose and galactose. The relative sharp bands at 765.601 and 722.211 cm^−1^ corresponded to the out-of-plane N–H vibration of fatty acid. The presence of S–S stretching vibration (disulfides) at a medium and sharp band (443.547 cm^−1^) is considered the fingerprint of *D. implexa*.  Table 2 summarizes the characteristic functional group assignments for the major peaks identified in the FTIR spectra.

From the above signals, we can make a preliminary assessment and have many visualizations of the groups of compounds in the extract: From the signals of the -OH bond and the -C-O bond, we can suggest that the extract may have the presence of polyphenol compounds—a class of organic compounds consisting of a hydroxyl group (-OH) attached to an aromatic hydrocarbon group, such as phenol (C_6_H_5_OH) and compounds with a similar but more complex general structure. In addition, there is probably a presence of flavonoid compounds with the basic structure of the 2-phenyl benzopyrane or flavane nucleus, consisting of two benzene Rings A and B linked to a six-membered Ring C; we can specifically represent compounds belonging to the flavone and flavonol groups based on the appearance of the signals of the carbonyl group bond (-C=O) and the signals of the ether bond (-C-O-C-) for visualization of the C ring of the two groups of compounds above.

### 3.3. Estimation of Secondary Metabolites: Quantitative Phytochemical Analysis

Seaweed is gaining traction as a source of valuable ingredients beyond just its nutritional content. Seaweed packs a punch of powerful molecules called polyphenols, which are known for their diverse health benefits such as antimicrobial, anti-inflammatory, antioxidant, anticancer, and antidiabetic activities. Consequently, researchers are diving deep into the potential of polyphenolic compounds, exploring their potential use in medicine, food, and cosmetics [38].

In the current study, quantitative phytochemical analyses were estimated. The total polyphenol content (TPC) of the algae extract was determined based on the standard gallic acid (concentration from 0 to 40 μg/mL) with a linear regression equation of *y* = 0.0287*x* + 0.0796 (*R*^2^ = 0.9989). The TFC of algae extract was determined based on the quercetin standard (concentration from 0 to 120 μg/mL) with linear regression equation *y* = 0.0010*x* + 0.0142 (*R*^2^ = 0.9912). Based on these standard curves, *D. implexa* had a TPC of 85.95 ± 1.21 mg GAE/g extract and a TFC of 245.6 ± 2.83 mg QE/g extract (Table 3).

In this present work, the total phenolic content of the *D. implexa* extract (85.95 ± 1.21) was in line with Dang et al., who showed that the great TPC levels were detected in the seaweed extracts of *Sargassum linearifolium, Sargassum vestitum, Sargassum podocanthum, Phyllospora comosa, Hormosira banksii,* and *Padina* sp. (48.13–158.82 mg GAE/g extract) [39]. In addition, the ethanol extract of *D. implexa* recorded a higher value than several results of other studies such as *Turbinaria conoides* 27.64 ± 1.69 mg GAE/g [21], *Liagora viscida* 0.32 ± 0.05 mg GAE/g, *Osmundea pinnatifida* 0.94 ± 0.06 mg GAE/g, *Porphyra umbilicalis* 1.69 ± 0.15 mg GAE/g [40], *Caulerpa racemosa* 56.70 ± 2.34 mg GAE/g [41], and *Caulerpa scalpelliformis* 55.40 ± 39.70 mg GAE/g [42]. Studies indicated that different algal species produce different phenolic compounds [43].

The total flavonoid compounds in brown algae are considered one of the most important components of natural antioxidants due to their essential roles in anticancer, antioxidant, antibacterial, antiallergic, antidiabetic, antiaging, anti-inflammatory, and anti-HIV activities. As shown in Table 3, the TFC of the *D. implexa* extract was in line with El Shafay et al. (2022) who reported that the great TFC levels were detected in the seaweed extracts of *C. elongata, J. rubens, P. pavonica,* and *T. atomaria* (69.7–374.1 mg QE/g crude extract) [44]. Noticeably, the result of present work showed greater value than several results of other studies such as *Dictyopteris membranacea* 120.00 ± 0.04 mg QE/g [45], *Padina durvillaei* 16.16 ± 2.87 mg QE/g, *Ulva lactuca* 10.22 ± 0.96 mg QE/g [46], and *Kappaphycus alvarezii* 15.54 ± 1.68 mg QE/g [47]. The differences observed between studies may be due to differences in environmental parameters such as pH, salinity, temperature, geographical location, and biological parameters, which may affect the production of secondary metabolites by these organisms [48]. Moreover, flavonoids exhibited anti-inflammatory, antihepatotoxic, and antiulcer effects as well as anticardiac death [48]. Furthermore, the advantage of flavonoid compounds is that they are nontoxic to the human body and safe for the environment [49].

Seaweed contains large amounts of polysaccharides, which are structural components of cell walls. Many soluble polysaccharides present in algae are associated with cholesterol-lowering and hypoglycemic activity, while water-insoluble polysaccharides are associated with reduced gastrointestinal transit time [48]. It is clear from Table 3, the polysaccharide content of the *D. implexa* extract (457.14 ± 14.43 mg GLUE/g extract) was lower compared to Huynh et al. (2013), who indicated that the great polysaccharide content level was found in the seaweed extracts of *Sargassum microcystum* (1090.00 mg GLUE/g extract) [50]. The quantity of polysaccharides extracted from brown seaweed species is demonstrably influenced by several factors, including the specific algal species itself, the chosen extraction method, and environmental variables [51, 52].

Agar in brown algae is known to have many biological activities, including antioxidant, anticoagulant, antiviral, and immunoinflammatory activities that may find their relevance in nutraceuticals, foods, pharmaceutical functions, and applications. From Table 3, the recovery efficiency of agar containing in the ethanol extract of *D. implexa* is 2.79 ± 0.41 (%). It can be seen that the recovery efficiency of agar of *D. implexa* was lower in comparison to previous studies; for example, Martínez-Sanz et al. and Nguyen et al. showed that the greater recovery efficiency of agar was detected in the seaweed extracts of *Gelidium sesquipedale* 10%–12% [53] and *Gracilaria tenuistipitata* (18.23%–32.97%) [54]. Reshma et al. have shown that the quality of algae collected, storage methods, and experiment time were not the same, directly influencing the agar weight. In addition, the amount of agar of algae was also affected by the collected location and the season in which the algae was collected, and the impact of the surrounding habitat, the nutritional environment, and the freezing and thawing process directly affected the amount of agar in the algal sample [55].

This current result indicated that the recovery efficiency of fucoidan of *D. implexa* (0.68 ± 0.01%) was in line with Fauziee et al., who showed that the recovery efficiency of fucoidan was detected in the seaweed extracts of *Turbinaria ornata* 0.65 ± 0.03%, *Sargassum polycystum* 1.16 ± 0.07%, and *Padina boryana* 1.59 ± 0.16% [56]. Fucoidan, a polysaccharide extracted from brown seaweed, exhibits solubility in hot water and acidic solvents. It demonstrates a wide range of biological activities, including anticoagulant, antitumor, antivirus, antioxidant, immune activation, and hepatoprotective properties, as documented in the literature [57]. However, it is crucial to note that the specific bioactivity of fucoidan is significantly influenced by its inherent chemical composition and structural characteristics. Variations in the algal species used for extraction, its geographical origin and growth conditions, the chosen extraction method, and even the analytical techniques employed (such as solvent concentration and extraction time) can all significantly impact the yield and bioactivity of the final fucoidan product [58, 59].

### 3.4. Antioxidant Activity Assay

#### 3.4.1. DPPH Radical Scavenging Activity

The antioxidant capacity of the *D. implexa* extract was determined based on the DPPH radical scavenging activity efficiency shown in Table 4. DPPH radical scavenging activity was proportional to the extract concentration and neutralization efficiency, meaning that DPPH scavenging activity was high when the extract concentration was high and vice versa. The highest free radical scavenging efficiency was 56.21 ± 0.84% at a concentration of 8000 μg/mL, and the lowest was 15.98 ± 0.55% at a concentration of 1000 μg/mL. In addition, the amount of antioxidant equivalent to acid gallic gradually increased from 6 to 16 μg/mL with DPPH scavenging activity from 35.08 ± 0.53 to 89.60 ± 0.74%.

Seaweeds are recognized for their production of phenolic compounds, which are believed to reduce oxidative stress [60]. The quantity of these compounds can exhibit variation between different seaweed species, potentially influenced by factors such as climatic conditions and the seaweed's stage of growth [61]. The ability of the extract to neutralize DPPH free radicals was also determined based on the IC_50_ value (scavenging capacity of 50%, the ability to neutralize 50% of free radicals) calculated based on the linear regression equation and compared to the acid gallic standard. The IC_50_ value of the extract was 6672.33 ± 71.49 μg/mL (*y* = 0.0061*x* + 9.2988, *R*^2^ = 0.989), and the IC_50_ of gallic acid was 8.74 ± 0.42 μg/mL (*y* = 0.0059*x* + 13.694, *R*^2^ = 0.9852). The results showed that the extract had the ability to neutralize DPPH free radicals about 763.42 times lower than gallic acid. This current result indicated that the DPPH scavenging activity (%) of the *D. implexa* extract (56.21 ± 0.84% at a concentration of 8000 μg/mL) was higher than the result of Shin and Kang who showed that the DPPH scavenging activity at 10,000 μg/mL concentration was detected in the seaweed extracts of *Dactylosiphon bullosus* (29.54 ± 3.62%) and *Sporochnus radiciformis* (40.60 ± 2.70%) [62]. The type of solvent used can impact the extraction of antioxidants from brown algae. Polar solvents are effective at extracting phenolic compounds, while semipolar solvents can extract a wider range of compounds, including phenolics, terpenoids, alkaloids, and glycosides. Nonpolar solvents, on the other hand, are better suited for extracting nonpolar compounds like waxes, lipids, and volatile oils [63].

#### 3.4.2. RP

**Figure 3:**
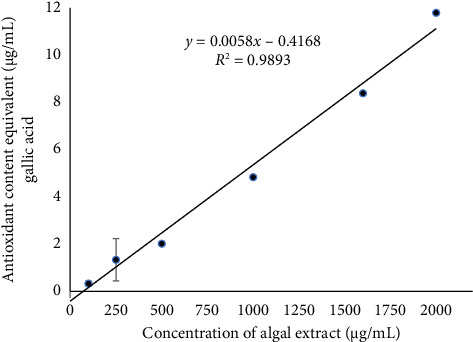
The gallic acid equivalent antioxidant content of the *D. implexa* extract.

The antioxidant effect of the *D. implexa* extract based on iron reduction ability was calculated as μg/mL of equivalent gallic acid based on the gallic acid standard curve equation: *y* = 0.0527*x* + 0.1745 (*R*^2^ = 0.9848). The results are presented in Figure 3.

Figure 3 shows that the antioxidant content in the algal extract equivalent to μg/mL gallic acid (iron reduction ability) was proportional to the extract concentration. The antioxidant content in the *D. implexa* extract equivalent to μg/mL of gallic acid increased from 0.32 ± 0.11 μg/mL at a concentration of 100 μg/mL to 11.79 ± 0.03 μg/mL at a concentration of 2000 μg/mL.

According to the present study, as the concentration gradually increased from 100 to 2000 μg/mL, the spectral absorbance of the algal extracts also gradually increased (0.19 ± 0.005 to 0.80 ± 0.002 nm), proving that the concentration of the extract was proportional to the spectral absorbance; the higher the concentration of the extract, the greater the spectral absorbance and vice versa. The free radical neutralization effect in the iron reduction method of *D. implexa* algae extract, and gallic acid was also investigated based on the OD_0.5_ value, indicating the concentration at which the sample or antioxidant can eliminate 50% of free radicals. Based on the gallic acid standard curve equation (*y* = 0.0527*x* + 0.1745, *R*^2^ = 0.9848) and the *D implexa* extract (*y* = 0.0003*x* + 0.1537; *R*^2^ = 0.99), the OD_0.5_ value of gallic acid and the algal extract were 6.18 ± 0.00 μg/mL and 1154.33 ± 0.00 μg/mL, respectively. This result showed that the antioxidant capacity of the *D. implexa* extract was about 186 times lower than that of gallic acid. Hwang et al. indicated that the hot water extract of *Sargassum hemiphyllum* showed the extract's OD_0.5_ value, which is the concentration needed to inhibit Fe^+3^ reduction by 50%, was found to be 408.33 μg/mL. The amount of antioxidant compounds can change depending on seaweed species and may be affected by climatic conditions and how old the seaweed is [61]. Moreover, different polarity of solvents can influence antioxidant activity [63].

### 3.5. Inhibition of Protein Denaturation

The anti-inflammatory activity of the *D. implexa* extract was investigated based on the inhibition of protein denaturation of BSA. Based on the results presented in Table 5, the algal extract's efficiency in inhibiting BSA denaturation increased from 45.38 ± 0.84% at the extract concentration of 5 μg/mL. The inhibition efficiency reached 69.93 ± 1.71% at the extract concentration of 40 μg/mL, and when the extract concentration increased to 100 μg/mL, the inhibition efficiency reached 103.64 ± 1.28%. From the current study, the ability to inhibit protein denaturation of the *D. implexa* extract (anti-inflammatory ability) was better in comparison to other algal extracts. The inhibition efficiency of *Plumeria rubra* reached 53.39 ± 0.00% at the extract concentration of 100 μg/mL [64], and the inhibition efficiency of *Ecklonia radiata* reached 35.46 ± 0.00% at the extract concentration of 600 μg/mL [65].

The anti-inflammatory ability of the *D. implexa* extract was compared to the diclofenac standard based on the extract concentration or the standard that inhibited 50% of protein denaturation (IC_50_, the half maximal inhibitory concentration). A low IC_50_ value refers to a high ability to inhibit the test sample's protein denaturation or strong anti-inflammatory activity. For bovine serum proteins, the IC_50_ values of *D. implexa* and diclofenac extracts were 9.95 ± 1.51 and 2.07 ± 0.38 μg/mL, respectively. It is clear that the ability to inhibit BSA denaturation of the algal extract was about 4.8 times lower than that of diclofenac. The results showed that the *D. implexa* extract could better inhibit protein denaturation (anti-inflammatory ability) than some other extracts. For example, *Plumeria pudica* extract reached IC_50_ at a concentration of 50 μg/mL [64], *Fucus vesiculosus* extract reached IC_50_ at a concentration of 200 μg/mL [66], and *Jania verrucosa* extract had IC_50_ at a concentration of 5000 μg/mL [65]. Many studies demonstrated a correlation between antioxidant activity, anti-inflammatory activity, and polyphenol content contained in the extract. The extracts with higher polyphenol content had higher antioxidant activity and stronger anti-inflammatory ability. Natural antioxidants are potential anti-inflammatory agents attracting attention in recent years [66, 67].

### 3.6. Antibacterial Assay

The antibacterial ability of the algal extract was determined based on its ability to inhibit bacterial growth, as shown by the diameter of the antibacterial ring created on the petri dish. The results showed that the extract at a concentration of 70 mg/mL had weak antibacterial activity against 2 bacterial strains *E. coli* and *B. cereus,* with antibacterial ring diameters of 9.00 ± 0.00 and 9.67 ± 1.16 (mm), respectively. The appearance of a sterile ring around the agar well may be due to the antibacterial active substances in the extract diffusing from the agar well to the surrounding agar surface and inhibiting the growth of bacteria.

The results of Table 6 show that the algae extract had a weak ability to inhibit the growth of 2 tested bacterial strains at concentrations ranging from 50 to 70 mg/mL. The level of resistance depended on the concentration of the extract used. In comparison to the inhibition ability on *E. coli*, the resistance effect of the extract was higher on *B. cereus* at a concentration of 70 mg/mL, and the resistance effect of the extract was lower than that of tetracycline at a level of 64 μg/mL, equivalent. The extract could not inhibit bacteria at 40 mg/mL concentrations against 2 tested strains. Vijayabaskar and Shiyamala found that extracts from two types of marine algae, *Turbinaria ornata* and *Sargassum wightii*, had noticeable activity against positive and negative bacteria [68]. The antibacterial ability of marine algae against microorganisms may change depending on season and reproductive state. Other important factors are the harvest period and the extraction protocol [69].

### 3.7. Cytotoxic Activities

**Figure 4:**
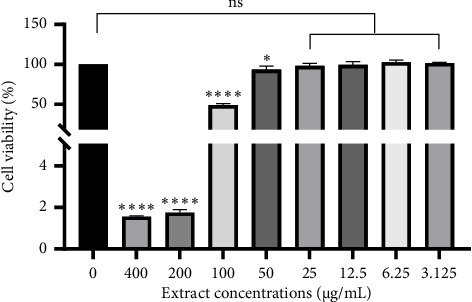
Cytotoxicity on HeLa cell line. Note: ns: no significance difference. Note: ^∗^*p*<0.05; ^∗∗∗∗^*p*<0.0001.

The ongoing pursuit of novel anticancer drugs from medicinal plants offers a real and encouraging path forward for cancer treatment [70]. Research suggests that extracts from various seaweeds exhibit cell-killing effects against diverse cancer cell lines [68–70]. The results of the in vitro anticancer activity using CKK-8 assay on HEK-293, HepG2, HT-29, HeLa, and Huh7 cell lines are presented in Table 7. The results revealed that the extracts showed potential cytotoxic activity against cancer cell lines at specific concentrations. Off these, the *D. implexa* extract displayed distinct cytotoxic effects on HeLa cells, with calculated IC_50_ values of 96.06 μg/mL. The results of toxicity testing of the extract on HeLa cells showed that the inhibitory effect on cell proliferation depends on the algal extract concentration; starting from a concentration of 3.125 μg/mL extract, the percentage of viable HeLa cells rapidly decreased and remained below 5% of viable cells when the extract concentration was greater than 200 μg/mL (Figure 4). Research indicated that brown algae are a rich source of polysaccharides, with fucoidan and laminarin being the most abundant types found within their cell walls [70]. These polysaccharides serve as the primary storage carbohydrates for these algae. Moreover, research has yielded promising results regarding the anticancer properties of fucoidan. In vitro and in vivo studies have demonstrated its potential effectiveness against various cancer types [71].

### 3.8. α-Amylase Inhibitory Activity

Oxidative stress and inflammation are the main causes of diabetes. Oxidative stress leads to increased blood glucose levels through a variety of pathways. Excess glucose also causes an increase in protein tyrosine phosphatase 1B (PTP1B) activity, which affects the insulin regulatory pathway. These processes are related to changes in redox status that cause Type 2 diabetes and its complications [72]. There are many associations between increased levels of acute-phase inflammatory markers and insulin resistance indices and the progression of Type 2 diabetes [73].

The ability to inhibit α-amylase was calculated based on the difference in the initial amount of starch and the amount of starch remaining after the hydrolysis reaction to evaluate the degree of hydrolysis of α-amylase. The more starch remaining after the reaction, the stronger the ability to inhibit α-amylase. Results from Table 8 show that the α-amylase inhibition efficiency increased linearly with increasing the extract concentration. The *D. implexa* extract had the highest inhibition at a 10,000 (g/mL concentration). In addition, the IC_50_ values for extracts of algae were 276.82 μg/mL, which showed lower inhibition effect in comparison to ethanol extracts of *A. nodosum* (IC_50_ 44.7 lg/mL), *F. serratus* (IC_50_ 70.6 lg/mL), *F. vesiculosus* (IC_50_ 59.1 lg/mL), and *P. canaliculata* (IC_50_ 51.0 lg/mL) [74] and higher than the potential of acetone extract of brown seaweed (*Spatoglossum schroederi*) for α-amylase inhibition (IC_50_ 580 μg/mL) [75]. Research has indicated that plant-derived phenolic phytochemicals have lower α-amylase inhibitory activity and a stronger inhibition activity against α-glucosidase [73, 74].

## 4. Conclusion

The current investigation has revealed that the brown algae, *D. implexa*, contain an abundance of secondary metabolites, including phenolics, flavonoids, and tannins. These findings suggest that D. *implexa* collected from Hon Son Island, Kien Giang, Vietnam, may be considered an outstanding natural source for developing therapeutic agents that can fight against oxidant, inflammatory, microbial, cancer, and diabetic. The prospect of identifying the specific bioactive compounds responsible for these observed biological activities serves as a continuous incentive for further research. Additionally, it is recommended that the applications of extracts from this species be further explored in *in vivo* studies to validate their potential for therapeutic and medicinal use. This study not only offers a potential solution to the environmental problem of excessive algae growth in coastal areas but also has the potential to stimulate economic growth by creating a novel method of producing pharmaceutical products from algae.

## Figures and Tables

**Table 1:** Biological activity of compounds in ethanol extract of *D. implexa*.

Compound	RT (min)	Area	%Area	Concentration (mg/g DW)
Quercetin	3.825	1685	1.029	0.524
Tannic acid	1.833	88,118	53.797	172.179

**Table 2:** Representation of wavelength and characteristic peaks of IR spectrum.

Sr. no.	FTIR peaks (cm^−1^)	Functional group assigned	References
1	3855.01	O-H stretching bend	[33]
2	3308.29	O-H stretching for alcohol and phenol	[34]
3	2924.52	N-H stretching, CH_3_ and CH_2_ stretching for aliphatic compounds	[35]
4	2856.06	C-H symmetry stretching (aliphatic)	[19]
5	1735.62	C=O, N=O stretching suggesting a sign of ester and amide groups	[36]
6	1661.37	C=O stretching, N=O asymmetric stretching (nitrate) for ester, pectin	[34]
7	1457.92	C-O stretching, O-H bending for cutin	[19]
8	1389.46	S=O, C-O, C-S, and C=S stretching resulting from the sulfate and phenols present in the seaweeds	[34]
9	1249.65	Broadband represents the sulfate group (S=O)	[34]
10	1154.19	Sulfate groups (S═O stretching) branching off from fucoidan or alginic acid residues	[37]
11	1082.83	Symmetric and asymmetric stretching vibrations of the RO─SO3- bond of the sulfate groups	[34]
12	938.199	The vibration of the C─O─C bridge of 3,6-anhydro-L-galactose and 3,6-anhydro-D-galactose (common to both agar and carrageenan)	[34]
13	887.095	Out-of-plane C-H bending for glucose, galactose	[19]
14	816.706	Out-of-plane C-H bending for glucose, galactose	[19]
15	765.601	Out of plane N-H wagging for fatty acids	[19]
16	722.211	Out of plane N-H wagging for fatty acids	[19]
17	682.677	C-S stretching, C=S stretching (sulfides) for sulfates	[19]
18	636.394	C-S stretching, C=S stretching (sulfides) for sulfates	[19]
19	443.547	S-S stretching for disulfides	[19]

**Table 3:** Phenolics, flavonoids, polysaccharides content, agar content, and fucoidan content in the *D. implexa* extract.

Seaweed	Phenolics (mg gallic acid equivalents/g crude extract)	Flavonoids (mg quercetin equivalents/g crude extract)	Polysaccharides content (mg glucose equivalents/g crude extract)	Recovery efficiency of agar (%)	Recovery efficiency of fucoidan (%)
*D. implexa*	85.95 ± 1.21	245.6 ± 2.83	457.14 ± 14.43	2.79 ± 0.41	0.68 ± 0.01

*Note:* Data are expressed as three replicates' mean ± standard deviation (SD).

**Table 4:** DPPH scavenging activity (%) of the *D*. *implexa* extract.

The concentration of the *D. implexa* extract (μg/mL)	DPPH scavenging activity (%) of the *D. implexa* extract	The concentration of gallic acid (μg/mL)	DPPH scavenging activity (%) of gallic acid (μg/mL)
1000	15.98 ± 0.55^g^	6	35.08 ± 0.53^f^
2000	20.36 ± 0.11^f^	8	46.94 ± 0.13^e^
2500	23.73 ± 0.07^e^	10	54.36 ± 0.13^d^
4000	33.52 ± 0.17^d^	12	66.53 ± 0.29^c^
5000	42.05 ± 0.48^c^	14	84.27 ± 0.16^b^
6250	49.61 ± 0.11^b^	16	89.60 ± 0.74^a^
8000	56.21 ± 0.84^a^		

*Note:* Data are expressed as three replicates' mean ± standard deviation (SD). Different letters in the same column indicate significant difference (*p* < 0.05).

**Table 5:** Inhibition of protein denaturation of the *D. implexa* extract.

Test sample	Conc. (μg/mL)	% protection
Ethanol extract of *D. implexa*	5	45.38 ± 0.84^f^
	10	51.26 ± 1.46^e^
	20	55.18 ± 3.5^d^
	40	69.93 ± 1.71^c^
	80	93.09 ± 0.58^b^
	100	103.64 ± 1.28^a^

Effect of diclofenac sodium (std. drugs)	5	50.70 ± 0.97^f^
	10	55.18 ± 0.49^e^
	20	61.35 ± 1.46^d^
	40	75.91 ± 0.97^c^
	80	98.32 ± 1.68^b^
	100	112.05 ± 1.94^a^

*Note:* Data are expressed as three replicates' mean ± standard deviation (SD). Different letters in the same column indicate significant difference (*p* < 0.05).

**Table 6:** Antibacterial circle diameter (mm) of the *D. implexa* extract and tetracycline.

Test sample	Conc. (mg/mL)	*Escherichia coli* (mm)	*Bacillus cereus* (mm)
Ethanol extract of *D. implexa*	40	0	0
	50	4.67 ± 0.58^c^	5.33 ± 1.16^b^
	60	6.33 ± 0.58^b^	7.67 ± 0.58^ab^
	70	9.00 ± 0.00^a^	9.67 ± 1.16^a^

	**Conc. (μg/mL)**		

Effect of tetracycline (std. drugs)	16	5.67 ± 0.58^d^	5.00 ± 1.00^d^
	32	8.33 ± 0.58^c^	8.67 ± 0.58^c^
	64	10.33 ± 0.58^bc^	11.33 ± 0.58^b^
	128	11.00 ± 0.00^b^	12.00 ± 1.00^b^
	256	13.67 ± 1.53^a^	14.67 ± 1.55^a^

*Note:* Data are expressed as three replicates' mean ± standard deviation (SD). Different letters in the same column indicate significant difference (*p* < 0.05).

**Table 7:** IC_50_ and selectivity index of the algal extracts on different cell lines.

Cell line	IC_50_ (μg/mL)	SI
HT-29	> 100	> 0.7
HeLa	96.06	0.72
Huh7	> 200	> 0.35
HepG2	> 200	> 0.35
HEK-293	69.88	1

*Note:* IC_50_ extract concentration is required to inhibit cell growth by 50% as obtained by CKK assay. Selectivity index (SI) indicates differential cytotoxicity of a compound (SI = IC_50_ treated normal cells/IC_50_ treated cancer cell lines).

Abbreviation: SI, selectivity Index.

**Table 8:** α-Amylase inhibitory activity of the *D. implexa* extrac**t**.

Concentration (μg/mL)	Inhibition (%)
10,000	92.85 ± 0.007
5000	51.53 ± 0.003
2500	40.97 ± 0.012
1250	27.88 ± 0.006
625	16.21 ± 0.003
312.5	6.24 ± 0.002

*Note:* Data are expressed as three replicates' mean ± standard deviation (SD).

## Data Availability

The datasets used in this study are novel and have not been disseminated publicly. Access to these data is granted through a request to the corresponding author.
